# Penile Length Assessment of Children Treated for Primary Buried Penis: Can Satisfying Penile Growth Always Be Achieved?

**DOI:** 10.3390/children10071254

**Published:** 2023-07-21

**Authors:** Marco Pensabene, Maria Sergio, Fabio Baldanza, Francesco Grasso, Gregorio Serra, Benedetto Spataro, Roberta Bonfiglio, Maria Patti, Valentina Maggiore, Chiara Cambiaso, Mario Giuffré, Giovanni Corsello, Marcello Cimador, Maria Rita Di Pace

**Affiliations:** 1 Pediatric Surgical Unit, Department of Health Promotion, Mother and Child Care, Internal Medicine and Medical Specialties, Piazza delle Cliniche, 2, 90127 Palermo, PA, Italy; marco.pensabene@policlinico.pa (M.P.);; 2 Neonatal Intensive Care Unit, Department of Health Promotion, Mother and Child Care, Internal Medicine and Medical Specialties, Piazza delle Cliniche, 2, 90127 Palermo, PA, Italy

**Keywords:** BP, children, micropenis, inconspicuous penis

## Abstract

Primary buried (BP) penis is describes as a small penis caused by a penile ligaments anomaly; it is unclear if a primary BP could reach a normal length. We selected 49 patients treated at our institution between 2015 and 2020 in order to post-operatively evaluate the SPL after one year. SPL was evaluated according to the PH Tanner staging system for pre-pubertal patients according to age-normalized values. A micropenis was detected if the SPL was below 2.5 SD. A normal SPL was found in thirty-two patients, eighteen were in PH Stage 1, four were in PH Stage 2, six were in PH Stage 3, and four were in PH Stage 5. Seventeen patients showed a reduced SPL; in seven of these (four in PH Stage 4 and three in PH Stage 5), their SPL was <2.5 ST. The difference in micropenis prevalence between the pre-pubertal and post-pubertal patients was significant (*p* = 0.038). A primary BP grows normally during the pre-pubertal period, where patients frequently showed a normal SPL, but it seems to be unable to reach a normal length in the higher PH stages, where the SPL is used to detect a micropenis. We suggest that a primary BP should be considered not as a simple defect of the penile ligaments and surrounding tissues, but as an incomplete manifestation of a micropenis due to a growth slowdown of the organ in late puberty.

## 1. Introduction

An inconspicuous penis is a general definition which includes a spectrum of conditions characterized by a diminutive aspect of male external genitalia. It could be caused by different congenital or acquired anomalies and includes a “concealed penis” and a micropenis. We know many variants of concealed penis such as a BP, in its congenital or acquired form; a webbed penis, a congenital anomaly of peno-scrotal angle; and a trapped penis, commonly secondary to a complicated circumcision. A congenital BP is an uncommon feature in which a normal-sized penis looks smaller than normal and is hidden in the pre-pubic tissue. More precisely, it was defined by Maizels et al. as “a congenital anomaly in which a normal-sized penis is hidden below the surface of prepubic skin” [1]. In 1977, Crawford proposed a classification which divided this condition into primary and secondary forms; the first one is caused by an incorrect or complete lack of anchoring of the superficial fascia and skin to the deep fascia at the base of the penis, resulting in an anomaly of the peno-pubic angle, with the penis buried in the pre-pubic tissue; the secondary form is caused by excessive suprapubic fat, especially in obese adolescents. In these cases, despite the penis appearing to be hidden in the pre-pubic fat, a normal peno-pubic angle can be found [2]. The primary form commonly needs surgical correction, while the latter one usually could improve with age, somatic development, or alimentary restrictions.

The BP, as well as all the concealed penis variants, can be distinguished from a micropenis on the basis of penile length [3]. While in the concealed penis, the aberrant anatomy of the penile ligaments and surrounding tissues is recognized, in the second one, a normal-shaped penis is shorter and smaller than expected, with a stretched penile length > 2.5 SD below the normal value. It is commonly observed in patients treated for hypospadias, especially proximal ones, or in cases of hypogonadotropic hypogonadism (in its primary or secondary form), or partial/mild androgen insensitivity syndrome (PAIS or MAIS) [3,4,5].

According to the literature, both primary and secondary BPs are expected to have a normal penile length.

However, many authors have speculated whether the primary BP could achieve normal growth or not with different, and sometimes opposite, results. To date, this topic still remains a matter of debate among pediatric urologists.

The aim of this study is to evaluate penile growth in patients treated for a primary BP.

## 2. Materials and Methods

We retrospectively evaluated all patients treated for a primary BP between January 2015 and December 2020 at the Pediatric Surgery and Urology Unit, Azienda Ospedaliera- Universitaria Policlinico“P. Giaccone”, Palermo, Italy.

Patient selection. The inclusion criteria were: (1) patients treated for an isolated primary BP; (2) the absence of surgical complications as major bleeding, dehiscence, recurrence, and retracting scars; (3) the treatment being performed by a single surgeon. Patients with a “webbed” or “trapped” penis were excluded, as well as patients with hypospadias or other conditions potentially leading to endocrine anomalies, or those with any potential cause for the penile growth impairment.

Penile length assessment. The penile length was assessed during post-operative examinations using the “stretched penile length” technique (SPL) after 1 year [3,5]; all data on penile length were reported in a specific database and collected for the present paper. The genital stage (GS) was defined by US measurement of testicular volume, from Stages 1 to 4, which represent the respective groups: from 1 to 3.9 cc, from 4 to 11.9 cc, from 12 to 20 cc, and larger than 20 cc [6]. The onset of puberty was defined as having a testicular volume greater than 4cc (GS 2). The “Pubic hair” (PH) stage was assessed according to Tanner and Marshall [6,7], from Stage 1 (the absence of pubic hairs—pre-pubertal status) to Stage 5 (full pubertal development). According to the literature, it has been demonstrated how the PH stage shows a stricter relationship with penile length if it is compared with age and the GS [6]. Consequently, in pre-pubertal patients (PH1 stage), the SPL was evaluated according to age-normalized values, while in the remaining patients, the SPL was evaluated according to the PH stage-related values [8]. Patients in PH5 older than 15 were considered as adults [5]. According to Aaronson, a micropenis was diagnosed if the SPL was below 2.5 SD [4]. In order to evaluate the prevalence of micropenises, we decided to compare patients on the basis of puberty onset; thus, patients in PH1 were compared to patients in PH2–5.

Surgical technique. A single surgeon performed all the surgical interventions, while another surgeon performed all the clinical examinations and PH staging assessments. In all cases, the surgical technique involved the positioning of an indwelling catheter, and local anesthesia was not used because the dissection of the surgical planes could have been complicated. The degloving of the penis was performed in order to remove all aberrant tissues; once the suspensory penile ligament was exposed, it was freed and the deep pre-pubic fascia was exposed. The fixation of the penis to the fascia was performed with 2-0 or 3-0 non-absorbable stitches. In this phase, care must be paid to avoid the neuro-vascular bundle of the penis, which enters the corpora at the 2 and 10 o’clock positions, as a lesion in this structure could compromise the patient’s erectile function.

The reconstruction of peno-pubic and peno-scrotal angles was performed with 4 stitches between the deep dermis of the skin and Buck’s fascia lateral to the posterior neurovascular bundle and lateral to the urethra [9].

In all patients, a compressive dressing was used; the indwelling catheter was left in situ, removed on post-operative day 5, and the patients were discharged after spontaneous micturition. Follow-ups were post-operatively performed on months 1, 6, and 12.

All subjects gave their informed consent for inclusion before they participated in the study.

Statistical analysis. Fisher’s exact test was used to evaluate the differences in the micropenis prevalence in pre-pubertal and post-pubertal patients, and a significance was identified with *p* < 0.05. The statistical test was identified as appropriate for the comparison of a single variable (micropenis/not micropenis) between two small populations.

Data were analyzed using software available online at “JavaStat” https://statpages.info/ctab2x2.html (accessed on 31 March 2023).

## 3. Results

From January 2015 to December 2020, 97 patients were operated on at our institution for an “inconspicuous” penis by a single surgeon. Forty-four of them did not satisfy the inclusion criteria and were excluded from the study: twenty-four had a “webbed” or “trapped” penis and a recurrent BP; eleven had hypospadias (five distal, four middle, and two proximal or peno-scrotal ones); four patients had cryptorchidism; two patients presented a neurological impairment (due to an intrauterine CMV infection); one patient had Currarino’s syndrome; one had glandular epispadias; and one had a monosomy of chromosome 18. Four of the remaining patients refused to be included into the study. Finally, 49 patients satisfied the inclusion criteria and were enrolled in this paper.

According to the genital stage, 15 patients were found to be in G1 (mean testicular volume: 2.1 mL; mean age: 4.03 years old; range: 2–9), 6 were in G2 (mean testicular volume: 7.8 mL; mean age: 8.75 years old; range: 8–11), 14 were in G3 (mean testicular volume: 17.15 mL; mean age: 12.8 years old; range: 11.5–14), and 14 were in G4 (mean testicular volume: 23.8; mean age: 15.9 years old; range: 13.8–18).

**Figure 1:**
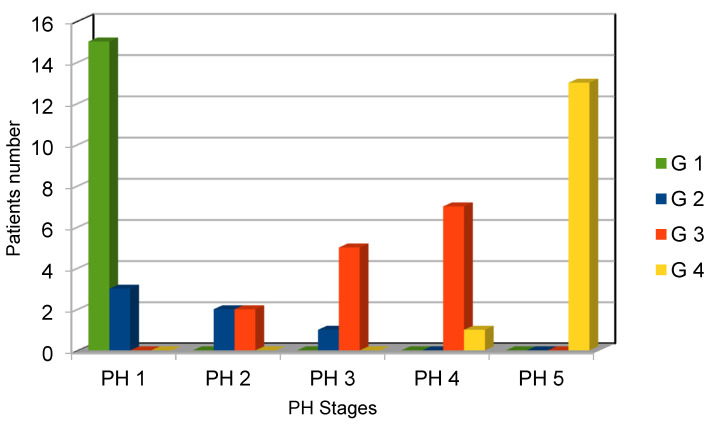
Relationship between genital and PH stage.

According to the pubic hair status, 18 patients were found to be in PH Stage 1 (36.75%; mean age: 4.4 years old; range: 2–9.5); 4 were in PH Stage 2 (8%; mean age: 9.7 years old; range: 9–11.2); 6 were in PH Stage 3 (12.25%; mean age: 12.6 years; range: 11–13.4); 8 were in PH Stage 4 (16.32%; mean age: 14.1 years old; range: 13.5–15);and 13 were in PH Stage 5 (26.55%; mean age: 16 years old; range: 14.5–18). The connections between the genital and PH stages are shown in Figure 1. The mean age at surgery was 5.3-year old (range: 13 months–10.8 years old), while mean follow-up period was 43 months (range: 13.5–94 months).

Major post-operative complications leading to the possible failure of the surgical procedure were not observed in any patients.

### Evaluation of SPL

Normal values of SPL were found in twenty-two patients (47.9%; 8.5 years old; range: 2–18), ten were in PH1, two were in PH2, six were in PH3, and four were in PH5. Ten patients (20.4%; mean age: 5 years old; range: 3–9.5), eight in PH1 and two in PH2, presented with an SPL below 1 SD. Nevertheless, we attributed minimal clinical significance to this finding, and these patients were assimilated into the normal group (Table 1). In the remaining seventeen patients (34.7%; mean age: 15.3 years old), eight in PH4 and nine in PH5, we found a short SPL (Table 1).

Ten patients (20.4%; mean age: 14.7 years old), presented with a short SPL (<2 SD): four patients in PH4 presented a mean SPL of 8.6 cm, while six in PH5 presented a mean SPL of 9.5 cm.

In seven cases, the SPL was below 2.5 SD, suggesting the diagnosis of a micropenis.

All eight patients in PH Stage 4 showed a short SPL; the overall mean SPL observed was 7.85 cm. There is a varied picture for patients in PH Stage 5: the SPL was suggestive for a micropenis in three cases, with a mean SPL of 7.2 cm (mean: 2.5; SD: 9.3 cm), and in six patients, we found a mean SPL of 9.5 cm, which is close to the micropenis limits. The results are summarized in Table 1 and Table 2.

Four patients in PH Stage 5 showed a normal SPL, with a mean value of 11.2 cm. Overall, in the PH Stage 5 patients, the mean SPL was 9.46 cm (Table 1).

In summary, we observed a short SPL in seventeen patients (34.7%), which were all found in the higher PH stages (PH Stages 4 and 5); seven of them, three in PH Stage 5 and four in PH Stage 4, had an SPL below 2.5 SD (Table 2).

In the remaining ten patients, six in PH Stage 5 and four in PH Stage 4, the SPL was close to the micropenis limits. All the remaining patients presented with an SPL within the normal values.

The prevalence of micropenises (SPL < 2.5 SD) was significantly higher in the post-pubertal patients than it was in the pre-pubertal ones (*p* = 0.038) (Table 3).

The mean SPL 2.5 SD reference values for PH4 and PH5 are, respectively: 8.475 cm and 9.3 cm.

## 4. Discussion

In the presented series, among the patients treated for a primary BP, we observed a more significant difference in prevalence of micropenis micropenises in the post-pubertal patients compared to that of the pre-pubertal ones. This difference is more evident in the patients at the higher PH stages and who have undergone complete or almost complete pubertal development. In fact, 7/31 patients who presented with a micropenis were in PH Stage 4 or 5. Moreover, in 10 patients, we found a short SPL, which was very close to the micropenis limit, even if it was not strictly over pathological range; once again, all these patients were in PH Stage 4 or 5. This finding suggests that penile growth could be impaired in patients with a BP, and it becomes more and more evident during pubertal development. At the same time, in four patients in PH Stage 5, a normal SPL was found. These findings seem to be unrelated to the surgical factors, as all the patients were treated using the same surgical technique, and no major post-operative complications showed up. This observation could imply an intrinsic difficulty in predicting whether the penis will achieve normal growth or not in these patients, and therefore, they need to undergo a long follow-up.

The aspect of male external genitalia has been identified as an important factor in determining a satisfying self-perceived body image in children and young boys. The psychosocial impact of an uncorrected “inconspicuous penis” is potentially devastating, as many authors underlined [5,10]; in rare cases, children and young boys left untreated experience depression and anxiety [3,11,12]. Unfortunately, misdiagnoses and delayed or missed diagnoses are still common, especially in the case of BPs. Usually, patients are referred to a pediatric urologist for the correction of aesthetical issues or for the revision of a previously not indicated circumcision. In fact, a circumcised BP will still look uncircumcised after surgery [3].

Therefore, an increasing interest in concealed penises and true micropenis has been observed among physicians, with the subsequent refinement of different operative techniques and classification systems [3,9,13,14,15,16,17,18,19,20].

The key points of common techniques described for BP correction are: the reconstruction of penile ligaments inserted in the corpora; the restoration of the peno-pubic and peno-scrotal angles, and, in some cases, the excision of excessive supra pubic fat. Nevertheless, by extending the classic concept of hypospadias surgery to BPs as well, a large number of techniques described could underline the difficulty in finding the gold standard technique.

In this scenario, the evaluation of penile length is crucial. Many authors investigated the adequacy of measuring the penile length of concealed penises, especially for BPs. Although, the results are controversial and sometimes conflicting. Some authors reported normal SPLs, while others reported low SPL values, especially in adolescents [14,21,22,23,24]. Moreover, papers including a rigorous inclusion criteria, follow-ups, and SPL evaluations are lacking.

In 2000, Frank questioned, even provocatively, the low prevalence of these pathologies in the adult population, surmising the spontaneous resolution of these conditions during puberty [21]. Moreover, Radhakrishnan et al. [22] and Donahoe et al. [23] observed the normal sizes of glans and corpora cavernosa in their patients.

On the contrary, in 2005, Redman et al. reported a short “mean penile length” in his series composed of 31 patients with “buried” penises [24]. In this study, the author enrolled patients from 2 to 28 months of age with a mean age of 12 months and performed an evaluation of the SPL using the mean value, which was globally calculated for the entire cohort of patients.

In 2014, Hadidi published an interesting paper proposing a new classification system for BPs based on the worsening impairment of penile ligaments and the surrounding tissues, with the subsequent implication of a surgical strategy, thus finally identifying three BP grades [13]. This series is composed of 61 patients with a mean age at surgery of 15 months (range 6–48) and a mean SPL of 3.3 cm (range: 2.9–4.5); among the patients, the author included patients with epispadias or severe hypospadias.

At the same time, Hadidi reports the suggestive evaluation of the glans-to-penile ratio. The author reports a “reduced” (1:1) ratio in treated patients and noted that this ratio did not improve after a 10 year follow-up. A limit of this paper, according to the author, is that only 5 out of 61 patients underwent a valid follow-up period. Moreover, the author included patients with severe hypospadias and epispadias, possibly resulting in a confounding factor in the evaluation of the SPL, as in these conditions, penile growth could be impaired. Furthermore, the author did not perform the stratification of patients according to their ages or pubertal stage.

In 2020, Manasherova D. et al. described the Midline Incision Rotation Flaps (MIRF) technique [18]. The aforementioned technique involves a midline ventral incision, allowing the complete degloving of the penis, the removal of excessive pre-pubic fat, and the reconstruction of the peno-pubic and peno-scrotal angles.

In this paper, the author reports 18 patients treated using the MIRF technique, with a mean follow-up of 56 months (range: 36–72) and a mean age at surgical intervention of 1.4 years old. The evaluation was performed by a surgeon during the post-operative follow-up, up to 6 years post-operation, and the results were stratified into “good”, “satisfactory”, or “not satisfactory” groups. In 89% of the cases, the author reports “good” results in terms of penile length and the appearance of the genitalia. Nevertheless, in this paper, the author reports the subjective assessment of penile length, which should be evaluated using the SPL standard technique and compared to age-normalized values.

Recently, Wahyudi et al. reported a retrospective analysis of 133 cases treated with penile degloving and the complete excision of aberrant dartoic tissues, but without penile anchoring, reporting results after 6 months of follow-up [19]. The authors present a quite heterogeneous study population in this retrospective analysis, including patients ranging from <3 to 35 years old, but mostly (95.5%) between 3 and 18 years old (mean: 12). The results were obtained from questionnaires on post-operative satisfaction according to the patients or parents and surgeons. The post-operative outcomes were “very satisfying” or “satisfying” in most cases. The satisfaction effect in terms of size, shape, and voiding function was higher among the surgeons than it was among the patients, underlining the higher expectations in such patients, especially in terms of penile lengthening. Obesity and a constriction ring were commonly reported, respectively, at 39.8% and 34.6%, suggesting the possible inclusion of patients with a secondary BP or those with trapped penis in the series. The authors underline the role of obesity in post-operative complications, especially a prolonged post-operative edema (more than six weeks). A post-operative 3 cm mean increase in the SPL (range: 0.5–7 cm) was reported. The wide range of penile lengthening reported could be related to the heterogeneity in the population, ages, and PH Stage, making it difficult to compare these patients with ours.

We believe that the analysis of SPL using a single mean value in a poorly homogenous population with a wide range of age could represent a limit for the proper and reliable evaluation of SPL, as well as the simple glans-to-penile ratio. Furthermore, the subjective judgment of genital appearance in the post-operative period could represent an immediate tool for surgeons to evaluate the surgical results, especially in a small series of patients, but it should not be considered as a standard rating because of the intrinsic lack in reproducibility of any subjective estimation.

The reliable assessment of penile growth should be more precisely performed comparing SPLs with the age-normalized values for pre-pubertal patients and with the available “Pubic Hairs” stage-normalized values of patients who reached puberty, as PH staging seems to offer a more coherent relationship between the micropenis limits if they are compared both with the GS or age [6].

Nevertheless, we are aware that it is extremely difficult to obtain a consistent population of patients treated for BPs because of different factors. First, there is apparently a low prevalence of primary BPs, and, as cited above, frequent misdiagnoses or delayed/missed diagnoses. Furthermore, a certain number of patients could be excluded from a proper diagnosis because of psychosocial and emotive reasons; in the presented series, for example, four patients refused to be included in the study.

The wide range of ages involved in some studies, moreover, could represent a limit in the analysis of SPL and the interpretation of the results. In the presented series, for example, the patients ranged from 2 to 18 years old. We believe that, in order to overcome this limit, the stratification of patients according to age or to PH stage should always be performed.

The difficulty in diagnosis leads to general confusion regarding terms and definition, finally concurring with a difficulty in obtaining a wide and homogenous population. Terms such as “inconspicuous”, “concealed”, “hidden”, “buried”, and “trapped” penises are often erroneously used as synonyms, leading to frequent misdiagnosis, as mentioned above; moreover, primary and secondary forms of BP are frequently confused. As the secondary form usually can improve after puberty onset or alimentary restrictions, the primary form commonly requires surgical correction. It could determine a delayed diagnosis or undertreatment, with reported difficulty in engaging in sexual intercourse [18].

In 2015, Cimador et al. reported an exhaustive review, clearly reporting the definitions, anatomy, pathologies, and treatments of all conditions leading to an inconspicuous penis [3]. According to the authors of this study, in order to clarify the terminology, we suggest that the term “Inconspicuous penis” should be used as a general definition, which includes the “concealed” penis and the micropenis; the first one could be distinguished from the second one on the basis of the evaluation of SPL, with it being normal in the concealed penises and reduced in micropenises. Buried, webbed, and trapped penises should be considered as different variants of the concealed penis, as a normal SPL is expected to be reached in these cases.

A different consideration should be made for micropenises. According to Aaronson, a micropenis is suspected when a normally shaped penis is shorter than expected, more precisely, when the SPL is 2.5 SD lower than the normal values [4]. It may develop idiopathically or as a consequence of hormone asset and regulation anomalies, as commonly seen in Klinefelter syndrome, in PAIS/MAIS, in hypogonadotropic hypogonadism, and also in Androgen Receptor mutations [3].

Nevertheless, reduced values of SPL are commonly reported in patients treated for a primary BP.

Our results suggest that in these patients, a reduced SPL is more frequently encountered in the higher PH stages when compared with those of younger or pre-pubertal patients. A significant difference was observed in the prevalence of micropenis in post-pubertal vs. pre-pubertal patients.

In healthy boys, penile growth could be distinguished in two different phases, which are, respectively, before and after pubertal onset [25,26,27,28]. In the first phase, the penis shows a linear growth that follows somatic development, while in the second one, due to the action of increasing blood androgens, boys undergo on the rapid development of pubic hairs, and an acceleration in penile growth, with an exponential dimensional increase, which is observed especially between the PH1 stage and PH2 stage [27,28]. The penis reaches the definitive length once pubertal development is achieved (PH5 stage), usually between 16 and 18 years old. It is interesting to note, in our results, how the higher the PH stage is, the higher the incidence of SPL being below 2.5 SD and the micropenis limits is. We suspect that, in patients with a “BP”, penile growth can follow a normal trend pre-puberty, but a delay or impairment of growth seems to occur during puberty. This apparent penile growth slowdown could determine the pathological values of SPL. Consequently, in these patients, a long follow-up should be beneficial, as the early detection of penile growth impairment could ensure that they undergo proper androgen stimulation therapy. It has been shown, in fact, that androgen therapy increases its effectiveness when it is administered before full pubertal development. The early diagnosis and treatment of patients with an isolated idiopathic micropenis is, therefore, crucial [29,30].

According to the presented findings, we suggest that a primary BP should be considered as not only an anatomic defect of the penile ligaments and surrounding tissues, but also a complex condition due to both defective penile anatomy and development. It might be considered as an incomplete micropenis: a penis that is able to ensure normal development before puberty, but in some cases, it is unable to achieve normal exponential growth during puberty.

Limitations of the study. We are aware that the presented study simply underlines the increased prevalence of micropenis in this study-specific population. The next step, then, should be to investigate the hormone assets in all patients with a primary BP diagnosis in order to exclude undetected anomalies in the hypothalamic–pituitary–gonadal axis, or defects in androgen secretion or action. This investigation was not feasible in this retrospective study, and it could reduce the strength of our conclusions. Second, despite our attempts and selection criteria, it is challenging to obtain a homogeneous population of patients treated for a primary BP. This is due to different factors: frequent misdiagnoses or delayed diagnoses; proper diagnoses can also be “avoided” by patients themselves because of personal reasons, and this occurred in four cases in the presented series. Third, despite the fact that statistical analysis underlines significant differences in micropenis prevalence in pre- and post-pubertal ages, the presented data should be validated with prospective studies including wider and homogenous populations in order to confirm or exclude our conclusions.

## 5. Conclusions

In conclusion, a primary BP should be always considered in differential diagnoses of male genital malformations in order to reduce misdiagnoses, unnecessary circumcisions, and delayed diagnoses. Penile growth in primary PB patients can be impaired, but this finding could be unclear until pubertal development is complete or near complete. Therefore, penile length should be carefully investigated in these patients until full pubertal development is achieved; the early diagnosis of a micropenis, in fact, could ensure that the patient undergoing multidisciplinary evaluation and proper treatment when required. In unclear cases, patients should be soon referred to a pediatric urologist.

## Figures and Tables

**Table 1:** Patients are distributed according to the PH stage. Normal and reduced SPL patients are reported.

PH Stage	Patients	Normal SPL	SPL < 1SD	SPL < 2SD	SPL < 2.5 SD
PH1	18	10	8	/	/
PH2	4	2	2	/	/
PH3	6	6	/	/	/
PH4	8	/	/	4	4
PH5	13	4	/	6	3
		22 (44.9%)	10 (20.4%)		
Total	49	32 (65.3%)	10 (20.4%)	7 (14.3%)	

**Table 2:** Patients with reduced SPL are reported according to mean age, PH Stage, and SPL (mean).

SPL	Patients (Mean Age)	PH Stage	Patients (Number)	SPL (Mean, cm)
Reduced SPL	10 (14.7 years)	PH4	4/10	8.5 cm
		PH5	6/10	9.5 cm
Micropenis	7 (16.1 years)	PH4	4/7	7.1 cm
		PH5	3/7	7.2 cm

**Table 3:** Differences between pre-pubertal and post-pubertal patients with reduced SPL are reported.

	PH Stage 1	PH Stages 2–5	*p*-Value
SPL < 2 SD	0/18	17/31	0.000
SPL < 2.5 SD (Micropenis)	0/18	7/31	0.038

## Data Availability

The data presented in this study are available on request from the corresponding author. The data are not publicly available due to privacy reasons.

## Abbreviations

BP	buried penis
SPL	stretched penile length
PAIS	Partial androgen insensitivity syndrome
MAIS	Mild androgen insensitivity syndrome
GS	Genital Stage
PH	Pubic Hair (stage)
